# Epidemiology and Mortality of Surgical Amputations in Severely Injured Patients with Extremity Injuries—A Retrospective Analysis of 32,572 Patients from the TraumaRegister DGU^®^

**DOI:** 10.3390/jcm13227000

**Published:** 2024-11-20

**Authors:** Julian Scherer, Jakob Hax, Michel Paul Johan Teuben, Hans-Christoph Pape, Rolf Lefering, Kai Sprengel

**Affiliations:** 1 Orthopaedic Research Unit, University of Cape Town, H49 Old Main Building, Cape Town 7700, South Africa; 2 Department of Traumatology, University Hospital of Zurich, 8091 Zurich, Switzerland; jakob@hax.de (J.H.); michel.teuben@usz.ch (M.P.J.T.); hans-christoph.pape@usz.ch (H.-C.P.); 3 Department of Hip- and Knee Surgery, Schulthess Klinik, 8008 Zurich, Switzerland; 4 Institute for Research in Operative Medicine, University Witten/Herdecke, 51109 Cologne, Germany; rolf.lefering@uni-wh.de; 5 Praxis medOT, Hirslanden Clinic St. Anna, University of Lucerne, St-Anna-Str. 32, 6006 Lucerne, Switzerland; sprengelk@medot.ch

**Keywords:** trauma, amputation, severely injured patient, polytrauma, emergency medicine

## Abstract

**Background:** Extremity fractures are common injuries in polytraumatized patients. Concomitant injuries to the soft tissue, vessels and nerves in these fractures are defined as mangled extremities. The decision for or against limb salvage is dependent on the patient’s physiology and the limb status. In severely injured patients with critical physiological status, limb salvage may be contraindicated. International data on the epidemiology and management of mangled limbs in severely injured patients are lacking. Thus, the aim of this study was to assess the incidence of polytraumatized patients with severe injuries to either upper (UL) or lower limb (LL) as well as their management. **Methods:** A retrospective cohort analysis was conducted of patients aged 16 years and above with an Injury Severity Score (ISS) ≥ 16 who sustained fractures to the limbs and were admitted to a certified trauma center of the TraumaRegister DGU^®^ (TR-DGU) between 2009 and 2019. **Results:** In total, we assessed 32,572 patients (UL: 14,567, mean age 48.3 years, 70% male and LL: 18,005, mean age 47.0 years, 70.5% male) The mean ISS in UL was 28.8 (LL 29.3). Fractures to the humerus (*n* = 4969) and radius (*n* = 7008) were predominantly assessed in UL, and fractures to the femur (*n* = 9502) and tibia (*n* = 8076) were most common in LL. In both groups, the most frequent injury mechanism was motor vehicle accidents, and more than half (UL: 9416 and LL: 11,689) of the patients had additional severe Abbreviated Injury Scale (AIS) ≥ 3 chest trauma. 915 patients in UL and 1481 in LL died within 24 h of the index admission. Surgical amputation occurred in 242 (UL) and 422 (LL) cases with a peak ratio in patients with an ISS above 50 in both groups. In both groups, patients with severe concomitant chest trauma were more often surgically amputated. In both groups, fewer patients with surgical amputations died within 24 h of admission (3.3% vs. 6.3% UL; 6.4% vs. 8.3% LL) compared to patients without amputation, but more patients with surgical amputations died within the overall hospital admissions (15.7% vs. 11.9% UL; 19.2% vs. 14.2%). In both groups, hemodynamical shock as well as the administration of Packed Red Blood Cells (PRBCs) were associated with a higher amputation rate. **Conclusions:** Surgical amputations after major trauma seem to be rare. Hemodynamical instability seems to play a key role in the management of mangled limbs. Patients with life-saving surgical amputation still have an increased overall in-hospital mortality.

## 1. Introduction

Trauma to the extremities is one of the most common injury patterns in orthopedic trauma surgery. Amputation following traumatic injury is the second most common reason for surgical ablation after complications following vascular disease [1]. However, due to surgical advances, amputation rates following trauma have steadily declined over the years [2]. When assessing extremity injuries, all structural systems (bone, soft-tissue, vessels, and nerves) must be examined. Extremities with injuries to at least three of those systems are considered mangled limbs [3]. Several well-established scores for evaluating mangled extremities, such as the Mangled Extremity Severity Score (MESS), have been incorporated into daily clinical work; however, none of these have shown to be sufficient tools in prospective decision-making for salvage and reconstruction or primary amputation [4,5,6,7]. In mangled limbs, several factors such as age, comorbidities, injury severity, injury mechanism, contamination, other injuries, and the patient’s pre-trauma status should be considered, in order to sufficiently make a decision for the primary amputation of the affected limb or for limb salvage procedures [8]. In physiologically stable patients with a mangled limb, the decision for limb salvage and reconstruction depends on the severity of soft-tissue injury sustained, as well as the neurovascular status [9]. In polytraumatized or physiologically critical patients, however, limb salvage may be contraindicated to preserve life over limb [10]. International data on the epidemiology of mangled limbs and their management are lacking and are mainly focus on lower limbs. Data from the American College of Surgeons Trauma Quality Improvement Program database showed that 45.1% percent of patients with severe lower extremity injury were primarily amputated [11]. A study from the TraumaRegister DGU^®^ (TR-DGU^®^), assessing patients with an Injury Severity Score (ISS) > 9, showed a total incidence of traumatic macroamputation amongst the assessed collective of 1.9%, with one third affecting the upper limb. 86.4% of cases underwent formal surgical ablation, whereas only 13.6% underwent limb salvage procedures [12]. However, data on the epidemiology and mortality of severely injured patients (ISS ≥ 16) is lacking. Thus, the aim of the present study was to assess the incidence of severely injured patients with sustained severe injuries to either upper or lower limbs, as well as the chosen initial treatment algorithm in these patients. Furthermore, the trauma mechanism as well as concomitant injuries and mortality in these patients were analyzed.

## 2. Materials and Methods

### 2.1. The TraumaRegister DGU^®^

The TraumaRegister DGU^®^ of the German Trauma Society (Deutsche Gesellschaft für Unfallchirurgie, DGU) was founded in 1993. The aim of this multi-center database is a pseudonymized and standardized documentation of severely injured patients. Data are collected prospectively in four consecutive time phases from the site of the accident until dis- charge from hospital: (A) Pre-hospital phase, (B) Emergency room and initial surgery, (C) Intensive care unit and (D) Discharge. The documentation includes detailed information on demographics, injury pattern, comorbidities, pre- and in-hospital management, course on intensive care unit, relevant laboratory findings including data on transfusion and outcome of each individual. The inclusion criterion is admission to hospital via emergency room with subsequent ICU/ICM care or reach the hospital with vital signs and die before admission to ICU. The infrastructure for documentation, data management, and data analysis is provided by AUC—Academy for Trauma Surgery (AUC—Akademie der Unfallchirurgie GmbH), a company affiliated to the German Trauma Society. The scientific leadership is provided by the Committee on Emergency Medicine, Intensive Care and Trauma Management (Sektion NIS) of the German Trauma Society. The participating hospitals submit their data pseudonymized into a central database via a web-based application. Scientific data ana- lysis is approved according to a peer review procedure laid down in the publication guideline of TraumaRegister DGU^®^. The participating hospitals are primarily located in Germany (90%), but a rising number of hospitals of other countries contribute data as well (at the moment from Austria, Belgium, China, Finland, Luxembourg, Slovenia, Switzerland, The Netherlands, and the United Arab Emirates). Currently, more than 38,000 cases from almost 700 hospitals are entered into the database per year. Participation in TraumaRegister DGU^®^ is voluntary. For hospitals associated with TraumaNetzwerk DGU^®^, however, the entry of at least a basic data set is obligatory for reasons of quality assurance.

### 2.2. Patient Collective

We included all patients aged 16 years and above with an ISS ≥ 16 who sustained fractures to the limbs and were admitted to a TR-DGU^®^ hospital between 2009 and 2019 (TR-DGU-ID: 2019-013) [13]. Additionally, available AIS codes for fractures of the upper and the lower extremity, as well as associated injuries to vessels and nerves, were required for inclusion [14].

For the upper extremity, the following codes: 750500.2, 750900.2, 751100.2, 751272.3, 752800.2, 752272.3, 753200.2, 752274.3, 752400.2, 752500.2 (bones), 720299.2, 720208.3, 720699.2, 720608.3, 720499.2, 720406.3, 720806.3, 730604.2 (vessels and nerves) were used.

For the lower extremity, the following codes: 853000.3, 854500.2, 854000.2, 854001.3, 854441.2, 854456.3, 857200.2, 857300.2, 857400.2, 858100.2 (bones), 820299.3, 820208.4, 820699.2, 820608.3, 820499.2, 820406.3, 820899.2, 820806.3, 830399.2, 830499.2, 830699.2, 830599.2 (vessels and nerves) were used.

Injuries were grouped into two groups: upper and lower limb. Epidemiological data, as well as fracture distributions, were analyzed for each group. We further assessed concomitant injuries, trauma mechanism and mortality as an outcome measure. Surgical amputations were analyzed for each group and stratified by ISS.

### 2.3. Statistical Analysis

SPSS statistical software (version 26, IBM Corp., Armonk, NY, USA) was used for the analysis. Numbers of cases, percentages, means, and standard deviations (SD) were provided. Differences in categorial variables were evaluated with a chi-squared test, and differences in metric variables we used the non-parametric Kruskal–Wallis test.

## 3. Results

### 3.1. Injuries to the Upper Limb

#### 3.1.1. Epidemiology

In total, we assessed 14,567 patients with fractures to the UL with a mean age of 48.3 years (ranging from 16 to 106, SD 20.1) and a male predominance of 70% (*n* = 53 missing). The mean ISS for the upper limb group was 28.8 (range 16 to 75, SD 11.8). The type of injury was blunt trauma in most of the cases (93.5%, *n* = 631 missing).

#### 3.1.2. Fracture Distribution

Of all of the assessed patients with injuries to the UL (*n* = 14,567), 34.1% (*n* = 4969) sustained a fracture to the humerus, 48.1% (*n* = 7008) had fractures of the radius, 26.0% (*n* = 3790) had fractures of the ulna, 6.8% (*n* = 993) showed carpal fractures, 12.8% (*n* = 1866) had metacarpal fractures and 6.2% (*n* = 904) had fractures of the fingers. Regarding fractures involving joints, 1.7% (*n* = 200) of the patients sustained fractures to the wrist and 3.1% (*n* = 449) to the elbow joint.

#### 3.1.3. Concomitant Injuries, Trauma Mechanism and General Mortality

Amongst all assessed patients in the UL group, 3.7% (*n* = 544) sustained additional soft tissue injury whereas only 0.7% (*n* = 95) presented with concomitant vessel injury. Less than half of the patients (*n* = 6323, 43.4%) sustained severe (AIS ≥ 3) concomitant injuries to the head, whereas more than half (*n* = 9416, 64.6%) suffered from severe concomitant injuries to the chest. Only 19.3% (*n* = 2815) sustained severe concomitant injury to the abdomen. The most common injury mechanisms were motor vehicle accidents (MVA) with cars (*n* = 3856, 26.5%), followed by high falls (*n* = 3584, 24.6%), and MVA with motor bicycles (*n* = 2982, 20.5%), with the data of 213 patients missing. Only a minority of the assessed patients died within 24 h of the index injury (*n* = 915, 6.3%), whereas 11.9% (*n* = 1737) died within the index hospital admission.

#### 3.1.4. Amputations

**Figure 1:**
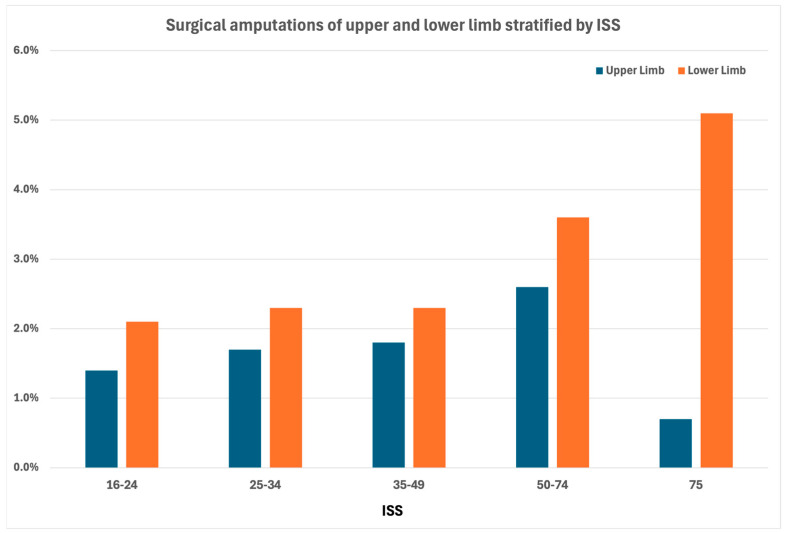
Occurrence of surgical amputations (%) of the upper and lower limb group stratified by ISS.

Surgical amputations occurred in 242 patients (1.7%), with the peak ratio in the ISS group between 50 and 74 (*p* = 0.046). (Table 1) A graphical comparison of the surgical comparison between upper and lower limbs stratified by ISS is given in Figure 1.

The most common injury mechanisms in the amputation group (*n* = 238, 4 missing) were MVA with a motor bicycle (45.0%), followed by MVA with a car (16%). 139 patients had a traumatic amputation, of which 52 (37.4%) were in addition surgically amputated. Most patients were amputated on the day of admission according to damage control principles (*n* = 186, 76.9%). Fewer patients with severe concomitant head injury were surgically amputated compared to patients without severe head injury (34.3% vs. 65.7%), whereas a different trend was seen in patients with severe concomitant chest trauma (62.0% vs. 38.0%). Less patients with surgical amputation died within 24 h of admission (3.3% vs. 6.3%) compared to patients without amputation, but more patients with surgical amputation died within the overall hospital admission (15.7% vs. 11.9%). Similar overall mortality was seen between patients undergoing surgical amputation within 24 h of admission (16.1%) and late amputation (14.3%) (*p* = 0.74). The mortality prognosis calculated with the RISC-II estimated higher mortality in patients with surgical amputation on admission (19.7%) than in patients with late amputation (18.9%).

Significantly more patients with subsequent surgical amputation presented with hemodynamical shock (blood pressure ≤ 90 mmHg) at admission (42.6%) compared to patients without amputation (16.1%) (*p* < 0.001).

The administration of packed red blood cells at initial in-hospital resuscitation was more often observed in patients undergoing surgical amputation (65.3%) compared to patients with no amputation (23.7%) (*p* < 0.001).

### 3.2. Injuries to the Lower Limb

#### 3.2.1. Epidemiology

The LL fracture group consisted of 18,005 patients with a mean age of 47.0 years (range 16 to 102, SD 20.1) and a male predominance of 70.5% (*n* = 69 missing). The mean ISS in the lower limb group was 29.3 (SD 12.2) and blunt trauma was sustained in 93.2% (*n* = 866 missing) of the cases.

#### 3.2.2. Fracture Distribution

Of all assessed patients with injuries to the LL (*n* = 18,005), 52.8.1% (*n* = 9502) sustained a fracture to the femur, 44.9% (*n* = 8076) had fractures of the tibia, 21.7% (*n* = 3899) had fractures of the fibula, 7.7% (*n* = 1386) showed patella fractures, and 20.8% (*n* = 3752) had foot fractures. Regarding fractures involving joints, 2.5% (*n* = 2445) of the patients sustained fractures to the knee joint and 2.6% (*n* = 469) to the ankle joint.

#### 3.2.3. Concomitant Injuries, Trauma Mechanism and General Mortality

Amongst all assessed patients in the LL group, 10.0% (*n* = 1792) sustained additional soft tissue injury, whereas only 1.9% (*n* = 345) presented with concomitant vessel injury. Less than half of the patients (*n* = 6638, 36.9%) sustained severe (AIS ≥ 3) concomitant injuries to the head, whereas more than half (*n* = 11,689, 64.9%) suffered from severe concomitant injuries to the chest. Only 21.1% (*n* = 3793) sustained severe concomitant injury to the abdomen. The most common injury mechanisms were motor vehicle accidents (MVA) with cars (*n* = 5670, 31.5%), followed by high falls (*n* = 3628, 20.1%) and MVA with motor bicycles (*n* = 3534, 19.6%), with the data of 254 patients missing. Only a minority of the assessed patients died within 24 h of the index injury (*n* = 1481, 8.2%) whereas 14.4% (*n* = 2585) died within the index hospital admission.

#### 3.2.4. Amputations

Surgical amputations occurred in 422 patients (2.3%), with the peak ratio in patients with an ISS of 75 (*p* < 0.001). (Table 2) A graphical comparison of surgical comparison between upper and lower limbs stratified by ISS is given in Figure 1.

Fewer patients with severe concomitant head injury were surgically amputated compared to patients without severe head injury (26.8% vs. 73.2%), whereas a different trend was seen in patients with severe concomitant chest trauma (58.5.0% vs. 41.5%). Less patients with surgical amputation died within 24 h of admission (6.4% vs. 8.3%) compared to patients without amputation, but more patients with surgical amputation died within the overall hospital admission (19.2% vs. 14.2%). There was a higher overall mortality in patients undergoing surgical amputation within 24 h of admission (20.1%) compared to patients with late amputation (15.5%) (*p* = 0.334). The mortality prognosis calculated with the RISC-II estimated a slightly lower mortality rate in patients with surgical amputation (23.1%) than in patients with late amputation (23.4%). Significantly more patients with subsequent surgical amputation presented with hemodynamical shock (blood pressure ≤ 90 mmHg) at admission (42.6%) compared to patients without amputation (19.5%) (*p* < 0.001).

The administration of packed red blood cells at initial in-hospital resuscitation was more often observed in patients undergoing surgical amputation (68.4%) compared to patients with no amputation (28.8%) (*p* < 0.001).

## 4. Discussion

The aim of this study was to assess the epidemiology of severely injured patients with upper and lower limb amputations, as well as the trauma mechanism, concomitant injuries, management and mortality. In this retrospective study of 32,572 (14,567 patients with injuries to the upper limb and 18,005 to the lower limb) severely injured patients, the rate of surgical amputations was relatively rare (1.7% vs. 2.3%) with the peak of both groups in patients with an ISS of more than 50. These findings are consistent with a large retrospective study from India reporting 2.5% major limb amputations after sustained trauma of the limbs [15]. However, they reported a 3-fold higher incidence of amputations of the upper limb, which is contrary to our findings and might be due to regional differences in trauma mechanism and surgical management. Unlike civilian trauma, battlefield trauma usually resulting from blast injuries as largely seen in the middle east as well as eastern Europe shows much higher amputation rates (13.6% UL, 17.4% LL). This might be due to a prolonged time in hospital, more severely injured extremities, and increased contamination [16]. Traumatic macroamputations at the scene were relatively rare, as reported by a previous study from the TraumaRegister DGU^®^ [12]. We assessed an increasing rate of traumatic as well as surgical amputations with increasing ISS and injury mechanism (MVA) in both upper and lower limbs. These findings are in keeping with previous studies reporting on the significant correlation of high-velocity trauma (MVA) and the resulting injury severity and the incidence of limb amputations [12,17,18]. Most of the assessed surgical ablations were performed in patients with an initial traumatic amputation according to damage control principles at arrival or on day one post-injury. Whether to attempt limb salvage or to amputate (irreparably) severely injured limbs is still a debate, happening alongside increasing surgical advances [3]. Previous literature has described the necessity of hemodynamical stability to attempt limb salvage procedures [19,20]. In contrast, hemodynamically unstable patients with mangled limbs should be amputated immediately to avoid further blood loss and the burden of salvage procedures on the patients’ impaired physiology, according to damage control principles (life over limb) [21,22]. Our data show that these principles were widely applied to this cohort. In both upper and lower limbs, more patients with hemodynamic shock and the use of PRBCs were amputated, which is further in keeping with the described surgical principles and decision-making in the management of mangled limbs. Additionally, more patients with chest trauma and fewer patients with injury to the head underwent amputation, which seems logical, keeping in mind that severe chest trauma has a greater additional effect on hemodynamic physiology and a need for PRBCs than intracranial trauma [23]. Furthermore, our data showed that surgical amputation on arrival in these patients increased the likelihood of 24 h survival, which might be due to the early control of exsanguinating limb injuries, and therefore successful (temporary) resuscitation [22]. However, the overall hospital-mortality was increased in patients undergoing surgical amputation, which seems contrary to the benefit of damage control principles. A possible explanation for this phenomenon might be the increased overall injury severity, resulting in the decision for surgical amputation and, consequently, an increased risk of mortality due to concomitant injuries [24]. In both subgroups, the RISC-II was overestimating the general mortality but predicted lower mortality in patients undergoing surgical amputation compared to patients without amputation, which is interesting since our data showed that hemodynamical instability was a factor in favor of surgical amputation, which is also included in the RISC-II variables. However, severe head injury, as a co-variable for the RISC-II calculation, was not a factor for surgical amputation as described above [25].

### Limitations

In general, the quality of registry data is considered inferior, due to it lacking data verification. Furthermore, this is a retrospective analysis, and thus only data from the registry itself were analyzed. In cases of a traumatic amputation, the operation code of a surgical amputation may not have been additionally recorded and was therefore not identified in this analysis. Furthermore, we could not assess further clinical management after the initial resuscitation phase (e.g., continuous bleeding, sepsis) which may impose a survivor bias in the non-amputation group. Finally, decision making could only be assumed depending on the registered variables such as outcome and performed amputation, and no verification of actual reasoning for the applied treatment strategy could be made.

## 5. Conclusions

Our data showed that surgical amputations after major trauma seem to be rare. Hemodynamical instability seems to play a key role in making the decision for or against mangled limb salvage. Patients with major trauma and thus subsequent life-saving surgical amputation still have an increased overall in-hospital mortality.

## Figures and Tables

**Table 1:** Occurrence of surgical amputations in the upper limb group stratified by ISS groups and ISS distribution. ISS arbitrarily grouped.

ISS	16–24		25–34		35–49		50–74		75		Total (*n*)
	*n*	%	*n*	%	*n*	%	*n*	%	*n*	%	
Surgical amputation	101	1.4	73	1.7	40	1.8	27	2.6	1	0.7	242
No surgical amputation	6908	98.6	4113	98.3	2133	98.2	1024	97.4	147	99.3	14,325
Total (*n*)	7009		4186		2173		1051		148		14,567

**Table 2:** Occurrence of surgical amputations in the lower limb group stratified by ISS groups and ISS distribution. ISS arbitrarily grouped. The most common injury mechanisms in the amputation group (*n* = 415, 7 missing) were MVA with motor bicycles (38.6%), followed by pedestrian accidents (17.1%). More than half of the patients (*n* = 281) had a traumatic amputation, of which 180 (64.1%) were, in addition, surgically amputated. Most patients were amputated on the day of admission according to damage control principles (*n* = 338, 80.1%).

ISS	16–24		25–34		35–49		50–74		75		Total (*n*)
	*n*	%	*n*	%	*n*	%	*n*	%	*n*	%	
Surgical amputation	174	2.1	123	2.3	61	2.3	54	3.6	10	5.1	422
No surgical amputation	8023	97.9	5324	97.7	2593	97.7	1458	96.4	185	94.9	17,583
Total (*n*)	8197		5447		2654		1512		195		18,005

## Data Availability

Data are available upon reasonable request.
